# Gender associated muscle-tendon adaptations to resistance training

**DOI:** 10.1371/journal.pone.0197852

**Published:** 2018-05-22

**Authors:** Gerard McMahon, Christopher I. Morse, Keith Winwood, Adrian Burden, Gladys L. Onambélé

**Affiliations:** 1 School of Sport, Ulster University, Belfast, United Kingdom; 2 Health, Exercise and Active Living (HEAL) Research Centre, Manchester Metropolitan University, Crewe, Cheshire, United Kingdom; University of Debrecen, HUNGARY

## Abstract

**Purpose:**

To compare the relative changes in muscle-tendon complex (MTC) properties following high load resistance training (RT) in young males and females, and determine any link with circulating TGFβ-1 and IGF-I levels.

**Methods:**

Twenty-eight participants were assigned to a training group and subdivided by sex (T males [TM] aged 20±1 year, *n* = 8, T females [TF] aged 19±3 year, *n* = 8), whilst age-matched 6 males and 6 females were assigned to control groups (ConM/F). The training groups completed 8 weeks of resistance training (RT). MTC properties (Vastus Lateralis, VL) physiological cross-sectional area (pCSA), quadriceps torque, patella tendon stiffness [K], Young’s modulus, volume, cross-sectional area, and length, circulating levels of TGFβ-1 and IGF-I were assessed at baseline and post RT.

**Results:**

Post RT, there was a significant increase in the mechanical and morphological properties of the MTC in both training groups, compared to ConM/F (p<0.001). However, there were no significant sex-specific changes in most MTC variables. There were however significant sex differences in changes in K, with females exhibiting greater changes than males at lower MVC (Maximal Voluntary Contraction) force levels (10% p = 0.030 & 20% MVC p = 0.032) and the opposite effect seen at higher force levels (90% p = 0.040 & 100% MVC p = 0.044). There were significant increases (p<0.05) in IGF-I in both TF and TM following training, with no change in TGFβ-1. There were no gender differences (p>0.05) in IGF-I or TGFβ-1. Interestingly, pooled population data showed that TGFβ-1 correlated with K at baseline, with no correlations identified between IGF-I and MTC properties.

**Conclusions:**

Greater resting TGFβ-1 levels are associated with superior tendon mechanical properties. RT can impact opposite ends of the patella tendon force-elongation relationship in each sex. Thus, different loading patterns may be needed to maximize resistance training adaptations in each sex.

## Introduction

The muscle-tendon complex (MTC) exhibits multiple physiological characteristics which differentially impact the physical capacities of males and females throughout the lifespan [1–3] with young, exercising females possibly more susceptible to overuse injuries (such as tendinopathies) than males [4].

In particular, there is increasing evidence of sex-specific chronic adaptations of the in-series elastic component to resistance training (RT), over and above the intrinsic gender differences in both the absolute MTC properties at rest, and its acute response to exercise. Indeed, differences in viscoelastic properties of the free tendon and tendon-aponeurosis between young males and females have been demonstrated [5–7], with females displaying lower stiffness, modulus, hysteresis and greater strain. The combination of data from previous studies [8, 9] highlight the difference in both resting and post-exercise tendon collagen fractional synthetic rate (FSR) between males and females, with FSR remaining significantly elevated 72 hours post 60 minutes leg-kicking, endurance type exercise in males. Further research has also shown that gender additionally influences the post resistance-exercise expression of tendon structural and extracellular matrix (ECM) regulatory components [10].

The sex difference in responsiveness to training in chronic response terms is highlighted in Westh et al.[11] who showed that long-term habitually trained young female runners displayed significantly lower tendon stiffness compared to similarly trained male runners. Interestingly however, these chronically trained female runners did not differ significantly in terms of tendon morphology or mechanical properties to female non-runners, which raises any questions with regards to any change in the intrinsic quality of the tendon with chronic training in females. It is also notable that sex differences have been demonstrated in the adaptability of MTC properties following an extended period of physical training in older individuals [12, 13], thereby emphasising the persistent nature of the superior responsiveness and adaptability in males. Whilst research strongly suggests that females demonstrate dissimilar relative adaption profiles to tendon mechanical stimuli compared to age-matched male counterparts, the adaptability and endocrine links associated with this observation following heavy load dynamic resistance training of tendon for instance, have yet to be elucidated.

*In vitro* work suggests of particular importance to the endocrine adaptation of tendon, are growth factors Transforming Growth Factor Beta– 1 (TGFβ-1) and Insulin-Like Growth Factor One (IGF-I). Their primary roles in tendon include proliferation and migration of fibroblasts, subsequently increasing the production of collagens and other extracellular matrix structures in these cells during the remodelling stages [14, 15]. In humans [16] direct administration of IGF-I enhanced the collagen fractional synthetic rate in young and older males. In parallel, administration of IGF-I plus TGFβ-1 together, significantly improved mechanical properties of rabbit tendon [17]. Recent work from Astill et al. [18] demonstrates that following an acute bout of RT, males and females both display significantly elevated IGF-I levels 3 hours post RT. However, only females had significantly elevated peritendinous levels of IGF-I at 4 hours post, whereas males did not. Additionally, males showed greater post-RT changes in Matrix Metallopeptidase 9 (MMP-9) levels than females, and females had a more prolonged exercise-induced elevations in tissue inhibitor of metalloproteinases-I (TIMP-I) than males.

Data shows no normative difference when comparing circulating TGFβ-1 levels in males, pre and post-menopausal females, and pregnant females [19, 20]. Additionally, to the authors’ knowledge, there is no evidence to show that in a young, healthy population, there are marked fluctuations in systemic TGFβ-1 levels [21]. At present, the literature on any link between the previously reported [22] acute *in vivo* TGFβ-1 response to mechanical loading, and the magnitude or nature of human MTC training adaptations is limited. A topical study is that of Heinemeier et al. [22] who found elevation in systemic TGFβ-1 levels (30%) following 1 hour of uphill (3%) treadmill running at 12kph in young persons, which the authors proposed may have been linked to the observed change in peri-tendon TGFβ-1 levels and hence regulation of Type I collagen synthesis. However, in this Heinemeier et al.’s study, the exercise protocol involved endurance running, and hence, arguably, a less than optimal training modality (compared to heavy resistance exercise) where the outcome aimed for is inducing MTC adaptations [23].

Taking the observed differences between sexes in MTC response/ adaptability to physical stimuli, no study to date has characterised the sex-specific adaptations of the MTC, following a period of heavy dynamic resistance training. In addition, it remains unclear whether any difference would be associated with major growth-factor candidates purported to influence MTC properties and adaptations to training. Therefore, the objectives of this study were to 1). Characterise the MTC adaptation to a period of dynamic, heavy-load resistance training in males and females, 2) identify any sex-related differences across MTC properties and 3) investigate whether any of the adaptive responses could be reflected in changes in two key circulating growth factors related to the MTC.

## Methods

### Participants

Twenty-eight young participants recruited from the local university campus, gave written informed consent to participate in the study. All procedures and experimental protocols were approved by the Manchester Metropolitan University Cheshire Campus ethics committee. Exclusion criteria included the presence of any known musculoskeletal, neurological, inflammatory, or metabolic disorders or injury. Participants took part in recreational activities such as team sports and had either never taken part in lower limb resistance training or had not done so within the previous 12 months. Each participant completed a physical activity diary, outlining that they each habitually completed 3–5 hours of non-resistance based moderate physical activity per week. Sixteen participants were then equally subdivided by sex and randomly assigned to a training group (T males [TM] age 20±1 years, mass 81±4Kg, T females [TF] age 19±3 years, mass 69±3Kg), whilst 6 males ([ConM] age 22±2 years, mass 82±2Kg) and 6 females ([ConF] age 23±4, mass 63±4Kg) were assigned to a control group (CON). All females were eumenorrheic (menstrual cycle duration of 26–30 days) and none used any form of Oral Contraceptive Pill, the latter having been shown to impact of the MTC properties in females [24].

### Study design

The study design was convenience sampling, with participants separated into groups according to sex followed by random allocation to one of two groups (i.e. training or control). Following familiarisation with laboratory procedures at least one week prior to testing proper, participants were assessed for MTC morphology/functional properties and growth factors at baseline (week 0). Measurements were repeated after 8 weeks resistance training (post-training).

### Muscle physiological cross-sectional area (pCSA)

The measurement techniques used for the calculation for physiological cross-sectional area of the *Vastus Lateralis* (VL) muscle in the current study have been documented elsewhere [25, 26]. Briefly, multiple anatomical cross-sectional area (aCSA) measures were made via brightness mode ultrasonography (7.5-MHz, 40mm array probe, AU5, Esoate Biomedica, Genoa, Itlay) at 25%, 50% and 75% along the length of the VL muscle (insertion to origin), together with pennation angles and fascicle lengths. Muscle volume was then calculated using the truncated cone method, which has been validated in a number of previous studies [27, 28]. VL pCSA was calculated by dividing muscle volume by fascicle length [28].

### Quadriceps torque

Maximal isometric knee extension torque was measured with the knee at 50° knee flexion (full knee extension = 0°) on the right leg of all participants. This angle was chosen to fall at 50% of the range of motion covered during the exercise routines, thereby minimising the effect of muscle length specificity on the reported muscle torque increments. After a series of warm up trials consisting of ten isokinetic contractions at 60°·s^−1^ at self-perceived 50–85% maximal effort, participants were instructed to rapidly exert maximal isometric force (maximal voluntary contraction, MVC) against the dynamometer (Cybex, Phoenix Healthcare, UK) lever arm. Joint torque data traces were displayed on the screen of a MacBook Air computer (Apple Computer, Cupertino, CA, USA), which was interfaced with an A/D system (Acknowledge, Biopac Systems, Santa Barbara, CA, USA) with a sampling frequency of 2000 Hz. Isometric contractions were held for ∼2 s at the plateau with a 60 s rest period between contractions. Peak torque was expressed as the average of data points over a 500 ms period at the plateau phase (i.e. 250 ms either side of the instantaneous peak torque). The peak torque of three extensions was used as the measure of torque in each participant.

### Estimation of co-contraction from electromyographical activity

A pair of Ag-AgCl electrodes (Neuroline 720, Ambu, Denmark), were placed on clean, shaved, and abraded skin, at 50% of femur length, in the mid-sagittal plane of the biceps femoris. The reference electrode (Blue sensor L, Ambu, Denmark) was placed on the lateral tibial condyle. The raw EMG signal was preamplified (MP100, Biopac Systems Inc., USA), amplified ×1000 (MP100, Biopac Systems Inc., USA), bandpass filtered between 10–500 Hz (Biopac Systems, USA) with a notch at 50Hz, and sampled at 2000 Hz. All EMG and torque signals were displayed in real time in AcqKnowledge software (Biopac systems Inc., USA) via a PC. Two maximal knee flexion contractions were carried out to obtain the EMG at maximal flexion torque. The root mean square (RMS) EMG activity was averaged for a 500ms period (average of 1.5ms moving windows) which coincided with the plateau of peak torque.

To reiterate, the EMG of the long head of the biceps femoris muscle was measured to ascertain the level of antagonist muscle co-contraction during the required isometric knee-extension performances. The biceps femoris torque during a knee-flexion contraction was calculated as described by McMahon et al. [25] whereby a linear relationship between BF EMG and torque is assumed, thus enabling the quantification of the ‘pull back torque’ during knee extensions, and ultimately, the total forces experienced by the patella tendon [29, 30].

### Tendon properties

The measures of tendon properties used in the current investigation have been described elsewhere [31]. Briefly, tendon elongation was determined using brightness mode ultrasound imaging over the apex of the patella in the sagittal plane, with the knee fixed at 90^0^ flexion as per the norm in *in vivo* tendon properties assessment. Five preconditioning trials were carried out to ensure reproducibility. Following this, three ramped, 6-second isometric contractions were monitored for the distance between the original position of the tissue under the skin, relative to the new position of the tissue using ultrasound images captured onto a personal computer at 25 Hz. The ultrasound output was synchronized using a square wave signal generator to allow temporal alignment with both torque and EMG data. Tendon displacement was determined at intervals of 10% of the maximal force (from 10% to 100%) using image J. Three efforts were analysed, and the average reported as the profile of tendon force versus elongation for the participant. The plotted force–elongation relationship was fitted with a second-order polynomial function, forced through zero. Instantaneous tendon stiffness (K) values were calculated from the slope of the tangents at 10% force intervals [30]. Mean tendon stiffness was the average stiffness value from 10–100% MVC.

Patellar tendon (PT) resting length (TL) and cross-sectional area (Tcsa) were also assessed with the knee joint at 90^o^ of flexion. TL was measured from the inferior pole of the patellar to the superior aspect of the tibial tuberosity determined from sagittal-plane ultrasound images. Tcsa was determined from the mean of transverse plane ultrasound images taken at 25%, 50%, and 75% TL. Young’s modulus (E) was computed as instantaneous stiffness multiplied by the ratio of resting TL/Tcsa. Mean tendon stiffness was the average stiffness value from 10–100% MVC.

PT volume (TVol) was calculated using the TL and Tcsa values along the length of the tendon using the truncated cone method, which used the same principles as those demonstrated on muscle volume assessments [27].

### Circulating growth factor levels (IGF-I and TGFβ-1)

Pre and post-training, following an overnight fast, (~10 hours), participants reported to the laboratory between 9-11am. 5 mL blood samples were collected from the antecubital vein of the forearm, placed in a crushed ice bed for 1.5 to 2-hours, and then centrifuged at 4°C for 10 min at 4,800 rpm, with the supernatant being removed and stored in at least two aliquots in eppendorfs at −20° Celsius for later analysis. IGF-I and TGFβ-1 were analysed using the standard enzyme-linked immuno-sorbent assay (ELISA) procedure, as described by McMahon et al. [31], with the overnight incubation for the first antibody binding phase option for the TGFβ-1 (thereby increasing the sensitivity of the readings). Post-training samples were taken 3–4 days post final training session, at the same time-of-day as the pre- training sampling for each participant.

The laboratory tests were timed to avoid diurnal variability or acute exercise-induced growth factor fluctuations.

### Resistance training

Resistance training (RT) was performed three times per week for 8 weeks at 80% of 1 repetition maximum (1RM) on the knee extensor complex. Exercises included the back squat, leg press, leg extension (Technogym, Berkshire, UK), lunge, Bulgarian split squat and Sampson chair. All exercise sessions were supervised by a member of the research team. Participants completed two familiarisation sessions at 70%1RM prior to commencing the resistance training program. 1RMs were measured at baseline and every 2 weeks, with loading weight progressed. Volume (i.e. repetitions and sets) was identical for each training group, with each training session consisting of four exercises and performing three sets of 10 repetitions per exercise for the first 4 weeks, and four sets of eight repetitions per exercise thereafter. Training sessions would typically last ~60 minutes, with training records being diligently completed during sessions.

### Statistics

Statistical analysis was carried out using IBM SPSS v19 (IBM Inc, USA). Data was analysed using a 4×2 ANCOVA with baseline measures used as covariates. The within-group factor was the phase of training (baseline, post-training) and the between-group factor was training group (i.e. TM, TF, ConM, ConF). Post-hoc comparisons are Bonferroni corrected and adjustments for multiple comparisons are applied in the correlation tables. All data are presented as mean ± standard error of the mean (SEM). Statistical significance was set with alpha at ≤ 0.05. Power (β) and effect size (ES) are reported for those changes that exhibited significant sex differences, where power was calculated post hoc using the independent t-test assumptions. The sample size required to identify sex differences in the morphological and mechanical properties of the MTC was deemed adequate given that achieved study power was ≥0.8.

## Results

### Sex differences at baseline

There were no significant sex differences (p>0.05) in pCSA, Tvol, Tcsa, TL, PT K, PT E, TGFβ-1 or IGF-I. As expected however, TM produced significantly (p<0.01) greater torque than TF.

### MTC properties changes

**Fig 1:**
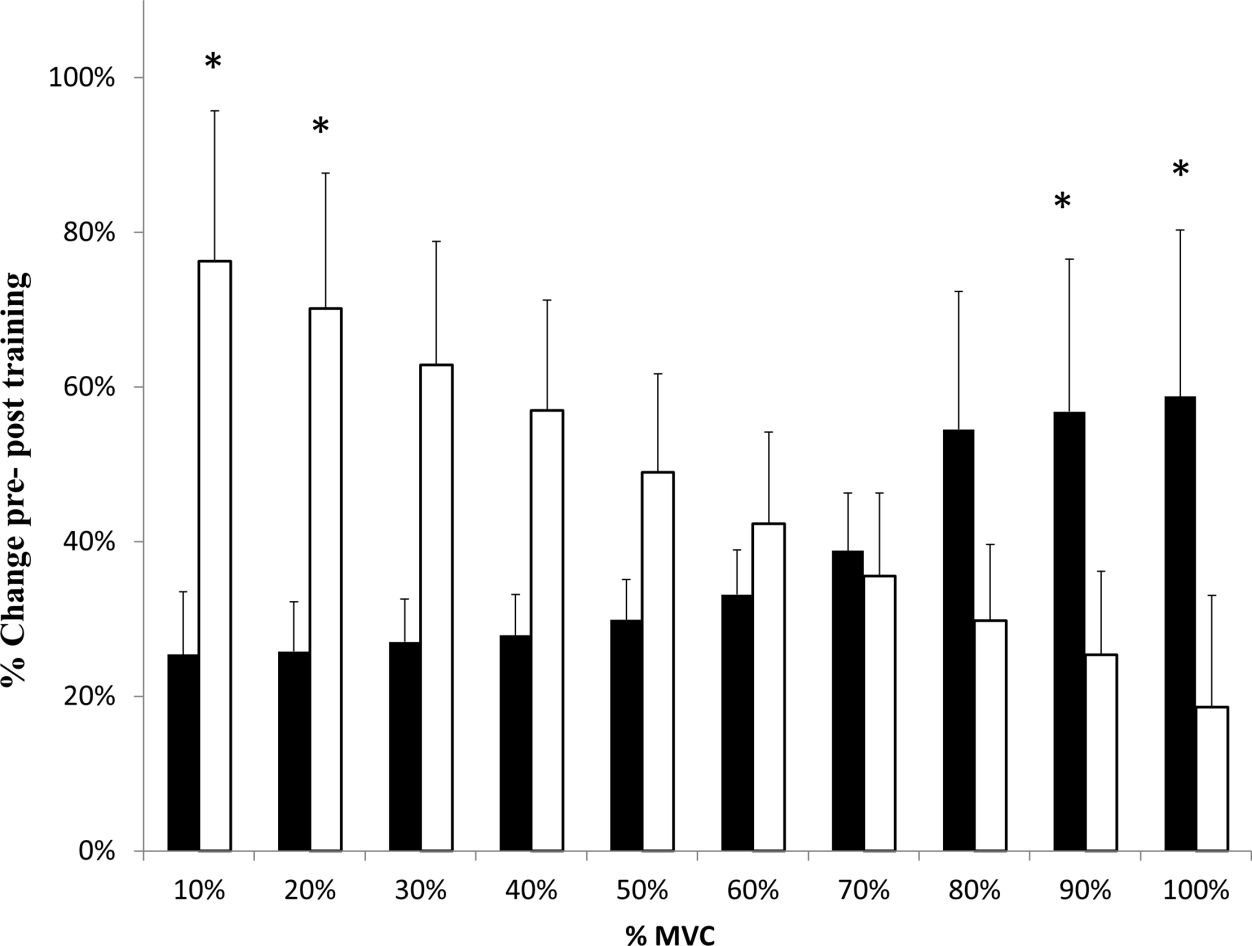
Relative changes in patella tendon stiffness (K) at each force level of the force-elongation curve. Males (black bars) and females (white bars) following training. * Significant difference (P<0.05) between sex. Data are Mean ± SEM.

There were significant increases in pCSA, strength, PT Vol, mean PT K and E, and IGF-I (Table 1) in each training group, with no sex differences. However, when PT K was analysed at discrete force regions, significant sex-specific differences were identified (Fig 1). TF had significantly greater increments in stiffness compared to TM following training at lower MVC forces (10% (p = 0.030, β 0.94, ES 0.29) and 20%MVC (p = 0.032, β 0.93, ES 0.28)), whereas TM had significantly stiffer tendons compared to TF at higher MVC forces (90% (p = 0.040, β 0.92, ES 0.24) and 100% (p = 0.044, β 0.79, ES 0.26)). There were no changes in TGFβ-1 in either the training groups (Table 1), or either of the control groups at week 8 (p>0.05). There was a significant increase in IGF-I in both male and female training groups post-training (p<0.05), however there was no differences detected between the groups (p>0.05, Table 1).

**Table 1:** Baseline and post-training values for muscle-tendon complex properties and circulating growth factors in each gender.

Measure	Males (*n* = 8)			Females (*n =* 8)		
	Baseline	Post-training	Change %	Baseline	Post-training	Change
**pCSA (cm**^**2**^**)**	71±4	87±7	24±9**	40±3	52±5	30±10**
**Strength (Nm)**	223±25	254±10	26±18**	145±11	177±10	25±6**
**T Vol (cm**^**3**^**)**	5.91±0.42	6.35±0.45	8±4*	4.10±0.24	4.38±0.32	6±2*
**Mean PT K (N mm**^**-1**^**)**	1132±104	1517±138	35±4**	619±72	887±108	46±11**
**Mean PT E (GPa)**	0.78±0.06	0.99±0.09	27±4*	0.42±0.04	0.60±0.08	46±16*
**TGFβ-1 (pg nL**^**-1**^**)**	5663±1524	4310±1248	-17±13	4716±1691	5276±2448	20±20
**IGF-I (ng nL**^**-1**^**)**	392±90	431±26	13±9*	380±24	460±42	23±9*

* Significant difference compared to baseline (p<0.05)

** (p<0.01). Data are Mean ± S.E.M. pCSA; Vastus Lateralis physiological Cross-sectional area, T Vol; Patella Tendon Volume, PT K; Patella Tendon Stiffness, PT E; Patella Tendon Modulus.

### Associations between IGF-I and TGFβ-1 against MTC characteristics

There was a significant positive correlation between mean tendon stiffness and TGFβ-1 levels (r = 0.554; p = 0.026) in the pooled population at baseline. Baseline pooled (not sex specific) TGFβ*-1* levels also correlated with baseline stiffness at some (30, 50 and 60% MVC) but not all torque levels (Table 2). Pooled population week 8 TGFβ*-1* levels correlated with baseline tendon stiffness at 10% through to 80% MVC. Pooled population baseline IGF-I values correlated with baseline stiffness at high force levels (i.e. from 60–100% MVC) as well as correlating with average stiffness. At week 8 however, the correlations of IGF-I was in fact with lower force regions (i.e. 10–50% MVC).

**Table 2:** Differential gender associations between circulating TBG-β, IGF-I and Tendon K.

%MVC		TGFβ-1 at Wk0			TGFβ-1 at Wk8			IGF-I at Wk0			IGF-I at Wk8		
		M	F	P	M	F	P	M	F	P	M	F	P
10%	Baseline K												
	Week 8 K												
	Δ K												
20%	Baseline K												
	Week 8 K												
	Δ K												
30%	Baseline K												
	Week 8 K												
	Δ K												
40%	Baseline K												
	Week 8 K												
	Δ K												
50%	Baseline K												
	Week 8 K												
	Δ K												
60%	Baseline K												
	Week 8 K												
	Δ K												
70%	Baseline K												
	Week 8 K												
	Δ K												
80%	Baseline K												
	Week 8 K												
	Δ K												
90%	Baseline K												
	Week 8 K												
	Δ K												
100%	Baseline K												
	Week 8 K												
	Δ K												
Average K	Baseline K												
	Week 8 K												
	Δ K												

Grey filled box denotes significant (p<0.05) correlation between circulating hormone level and force level variable in pooled (P), male (M) and female (F) populations. Δ (delta) K, change in stiffness.

Our sex specific observations showed that in males, baseline TGFβ-1 was associated with tendon stiffness at low forces (10–60% MVC). Post-training TGFβ-1 levels correlated significantly with post-training tendon stiffness at all force levels >30%MVC. Interestingly, TGFβ-1 levels post-training also correlated with baseline tendon stiffness, although at more moderate force levels (10–70%MVC). Whilst baseline IGF-I was not associated with tendon stiffness, at week 8, IGF-I levels correlated with week 8 stiffness at mid force levels (i.e. in the ranges of 20–60%MVC) as well as correlating with mean stiffness.

In contrast to the findings in males, females’ baseline TGFβ-1 did not correlate with stiffness at either baseline or post-training. Post-training TGFβ-1 levels only correlated with post-training tendon stiffness at 80–90% MVC. The only apparent/statistically significant relationships observed in females were with post-training IGF-I levels which correlated with baseline tendon stiffness at 40–70%MVC and tendon stiffness at all force levels (10–100% MVC) post-training.

## Discussion

Our key current findings are 1) we are the first to demonstrate sex-specificity in the overloading-induced adaptive nature of the mechanical properties of tendon in a young population. 2) Although TGFβ-1 and IGF-I levels may not reflect the entirety of adaptation magnitude, they still appear to play an important role in chronic MTC characteristics. 3) High-load, dynamic resistance training may not be optimal to enhance MTC characteristics in females at higher portions of the tendon force-elongation curve.

Sex-related differences in the mechanical, structural and regulatory mechanisms in human tendinous tissue have been identified previously [5, 7]. Differences in acute tendon fractional collagen synthesis rates [8, 9], amount of tendon dry mass per wet tendon weight [32], mRNA levels of Type III collagen [10] have all been shown to vary between sexes. In addition, proteomic work from Little et al. [33] demonstrated that alcohol dehydrogenase 1B, MMP-3 and thrombosponsin-1 (TSP-1) are enriched in female patella tendons compared to male tendons, which suggests perhaps a reduced extra-cellular matrix regulatory or remodelling function, and alteration of mechanical properties in females. This would tend to suggest that, either at rest, or when provided with a similar physical stimulus to males, female tendon does not respond similarly. As successive acute responses to physical stimuli combine to produce the chronic adaptation, this leads to the possible scenario of a mal-adapted female tendon (i.e. morphologically or mechanically) relative to male tendon following a period of training.

### Morphology and mechanical properties

**Fig 2:**
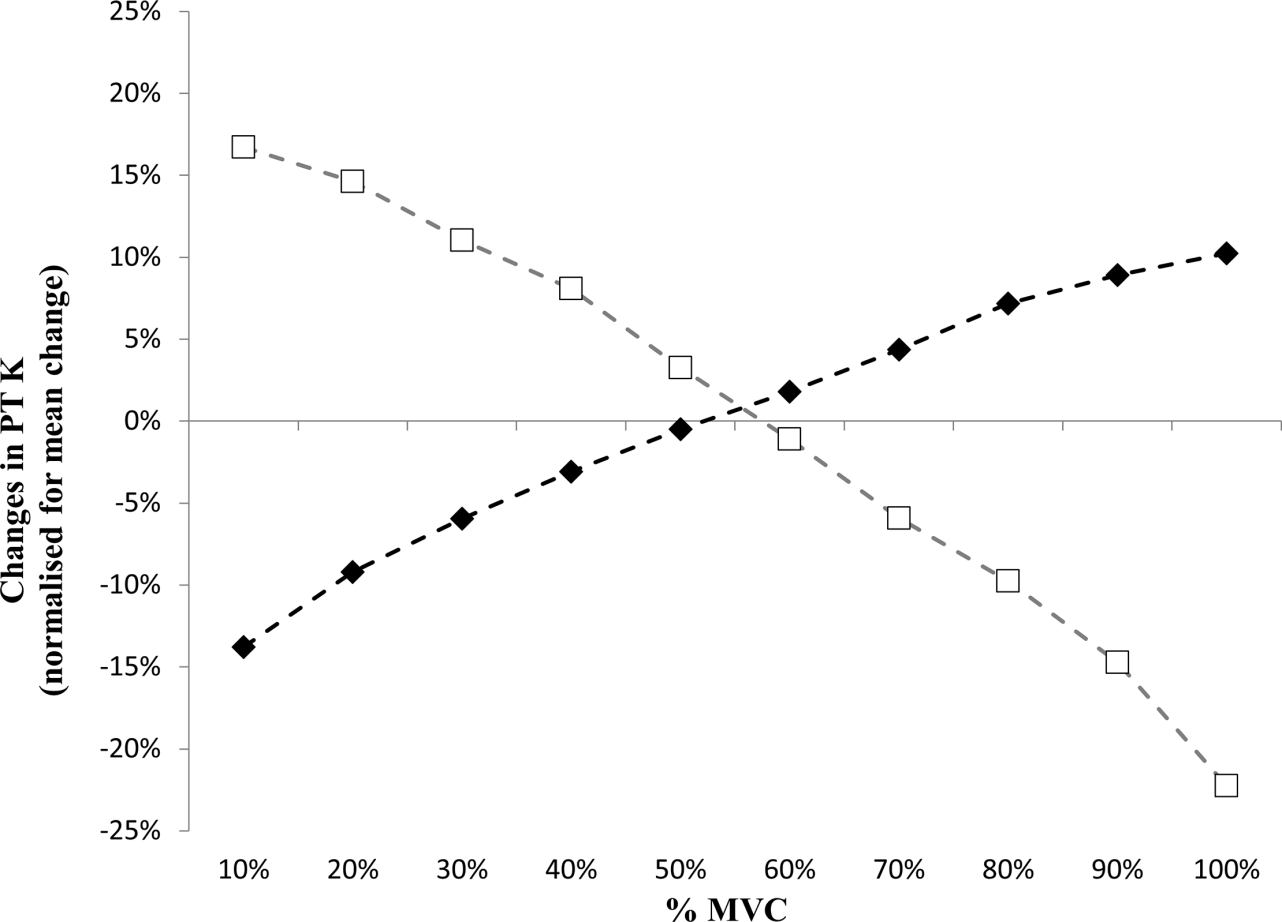
Normalized change in tendon stiffness to the mean stiffness change following training. Males (black filled diamonds) and females (white filled squares).

In the current study, we found that patella tendon volume significantly increased in both sexes by 8±4% and 6±2% in males and females respectively, with the small difference between sexes not being statistically significant. There were also no differences in the training-induced mean patella tendon stiffness change, between males and females. This is in contrast to the findings of Onambele-Pearson and Pearson [12] and Seynnes et al. [13] who found in older individuals (>70 years old), patella tendon stiffness changes in older males were significantly greater than that seen in older females following resistance-type and alpine skiing activities. What is interesting, in comparing the current investigation and the study of Onambele-Pearson and Pearson [12], is that both report the ‘character’ or ‘nature’ of tendon stiffness changes are inherently different between males and females following resistance training regardless of age. In their study, Onambele-Pearson and Pearson describe a ‘cut-off point’ of ~40%MVC, where below this juncture, females exhibited their greatest changes in stiffness, and above it, males displayed their greater changes in stiffness. In a similar fashion, we describe here for the first time in a young population, that at force levels of 10–20%MVC, and at 90–100%MVC females have a significantly higher or lower RT-induced stiffness change respectively than males, with a cut-off at ~55%MVC (Fig 2). Although not immediately apparent why, one plausible explanation is that the tendon adapts to the loading requirements it most frequently encounters, which in respect to males’ tendons, is a lower absolute load in females. Evident from the torque data in the current study, the mean torque associated with the resistance training would have been much greater in males (post-training MVCs were 254±10 Nm vs. 177±10 Nm in males and females respectively and were significantly different at baseline), despite both groups training at the same relative load (but distinct absolute loads).

### Resistance training program

A further potential physiological mechanism for our observations is the nature of the resistance training program. The exercises performed were dynamic (apart from one isometric), and isotonic in nature. Adaptations to eccentric training, such as microcirculatory factors and pain reduction, has been shown to be sex-specific in a cohort with Achilles tendinopathy, with males again demonstrating an improved responsiveness [34]. Furthermore, during maximal eccentric exercise between 20-90^o^ knee extension, we previously [7] showed that female patella tendon displayed reduced stiffness compared to males, and attributed a large portion of the reduced fascicular lengthening seen in females compared to males to this observation. A subsequent study from our group [35] also showed that following the same exercise protocol, males displayed a significantly greater magnitude of muscle damage. This demonstrates that the sex-specific response and adaptations to variables associated with manipulating a resistance training program have yet to be elucidated, and await further study. Therefore, the results of the current study raises the following question: Is dynamic, heavy-load resistance training (currently the most conventional and popular form), the best training method for females routinely operating at the higher force levels of the tendon force-elongation curve, where adaptations to this type of training are minimised relative to adaptations on the subsequent lower portions of the tendon Force-Elongation curve?

### Circulating IGF-I/ TGFβ-1

A further novel aspect of the current investigation was to ascertain the changes to circulating growth factors related to tendon and ECM responses/adaptation. *In vitro* studies have demonstrated the potency of TGFβ-1-mediated effects on collagen, and its relationship to magnitude of mechanical strain [14, 15]. Despite the vast array of data from *in vitro* studies outlining the roles of TGFβ-1 in tendon and ECM maintenance/ repair, data from *in vivo* human exercise studies is scarce. What is also surprising is that to date, only one study had previously described the effect of resistance training as opposed to endurance kicking-type exercise, on TGFβ-1 and tendon mechanical properties, despite resistance training being a more potent mechanical stimulus for tendon adaptation. We have also previously shown in a young population [31], that resistance training did not result in chronically elevated TGFβ-1 levels following 8 weeks of heavy resistance training with varying levels of strain. This was also the case in the current study, where there were no significant changes in either males or females following resistance training, despite significant improvements in tendon mechanical properties. Although a ~30% post-exercise increase in circulating TGFβ-1 associated with increased local Type I collagen synthesis and peri-tendinous TGFβ-1 levels has been documented [22], it may be that TGFβ-1 does not remain chronically elevated to maintain a healthy (non-fibrotic) connective tissue profile. Thus, within the confines of the current study’s experimental design, measurement of pre-and post-8 weeks of resistance training may not have been facilitative in uncovering TGFβ-1’s mechanistic role. Despite this, we have shown a strong, positive correlation between baseline TGFβ-1 levels and tendon stiffness. This would make sense, from the point that a natural higher physiological level of TGFβ-1 could produce and preserve a stiffer MTC. Previous research has shown, lower levels of TGFβ-1 and dysregulation of the TGF-β axis are present in diseased compared to healthy tendons [36]. It should nonetheless be noted that measuring TGFβ-1 in blood is a complex issue, with many large variations measured between studies. However, in a young, healthy population, such as in the current study, there have been shown to be no differences between male and females in circulating TGFβ-1 levels [19, 20]. Furthermore, our reported circulating TGFβ-1 levels are consistent with previously published data in a review using the same ELISA kit (R&D systems) and a robust TGFβ-1 sampling methodology [20].

IGF-I levels increased significantly as a result of heavy resistance training in both sexes. Similar observations have also been made, with peritendinous levels of IGF-I being significantly elevated at 3 hours post-RT in both males and females, but in females only, remaining elevated at 4 hours post RT [18]. In a patella tendon defect model in rabbits, direct administration of IGF-I and TGFβ-1 together significantly improved the mechanical properties of tendon such as force at failure, ultimate stress and stiffness [17]. Results from the studies of Doessing and co-workers [37] and Nielson et al. [16] demonstrate that IGF-I, and the regulatory function of the growth hormone/ IGF-I axis, are important factors for the matrix collagen fractional synthetic rate, expression of Type I collagen and tendon function. Circulating levels of IGF-I do not allow distinction between effects on muscle and/or tendon. In the current study, males and females showed very similar relative changes in IGF-I, muscle size, torque, tendon volume and tendon mechanical properties. IGF-I thus may have played a role in the adaptive process, with the work of Doessing et al. [37] suggesting that IGF-I may play a more prominent role in tendinous as opposed to muscular adaptation.

### Practical application

It has been suggested that young, exercising females are possibly at more risk of tendon injury than males [4]. The practical implications of the current study are that females operating toward the maximal end of the MVC spectrum, may experience a relatively reduced enhancement of tendon stiffness following RT. A sex-specific electromechanical delay has been noted previously [38], with females showing an increased delay compared to males. In parallel, we have also previously demonstrated that during maximal eccentric contractions [7], there are sex differences in absolute patella tendon stiffness, which in turn modulated *Vastus Lateralis* fascicle lengthening, affecting force production. These above facts compound the evidence and necessity to consider sex difference in resistance training applications. Therefore, female athletes involved in sprint/power activities may find that transfer of contractile force to bone during a high-force effort may be compromised in terms of amount of force and/ or rate of force development due to sub-optimal changes in stiffness, or indeed reduced force development due to modulation of the fascicular shortening. Therefore, future studies may wish to focus on elucidating methods to increase stiffness at higher MVC forces in young, female populations.

### Conclusion

In conclusion, we have demonstrated that both males and females display the same relative adaptability in terms of enhancing muscle-tendon morphology and function following resistance training. However, the nature of these adaptations have implications for muscle-tendon function during different tasks for each sex. Finally, for females, high-load dynamic resistance training may not be optimal for enhancing MTC function at high force outputs.

## Supporting information

S1 File Muscle-tendon male and female raw data results. (XLSX)

S2 File Endocrine growth factors regressions. (XLSX)
